# Extension agents’ attitudes and participation in disseminating climate-smart agricultural practices in North-Central, Nigeria

**DOI:** 10.3389/fnut.2025.1663720

**Published:** 2025-09-18

**Authors:** Ibukun Elizabeth Ojo, Ayorinde Ebenezer Kolawole, Abigail Gbemisola Adeyonu, Ayotunde Olayinka Owolabi, Dare Akerele, Toluwalase Eniola Awe, Ikechukwu Chike, Deborah Pelumi Ogunsuyi, Abisola Adeola Ogundele

**Affiliations:** ^1^Department of Agricultural Economics and Extension, Landmark University SDG 2 (Zero Hunger Group), Omu-Aran, Nigeria; ^2^Department of Agricultural Economics and Farm Management, Landmark University SDG 13 (Climate Action Research Group), Omu-Aran, Nigeria; ^3^Department of Agricultural Economics and Extension, Landmark University, Omu-Aran, Nigeria; ^4^Department of Agricultural Economics and Farm Management, Federal University of Agriculture, Abeokuta, Nigeria

**Keywords:** attitude, participation, CSAPs, extension agents, rice farmers, nutrition security

## Abstract

Low uptake of Climate-Smart Agricultural Practices (CSAPs) continues to exacerbate food insecurity and vulnerability in regions already burdened by poverty. CSAPs refer to agricultural methods that enhance productivity, climate resilience, and environmental sustainability. The effectiveness of extension agents is critical in promoting these practices, and their inefficiency can significantly weaken community resilience against hunger and environmental shocks. This study investigates the attitudes and participation of agricultural extension agents in disseminating CSAPs among rice farmers in North Central Nigeria. A multistage sampling procedure was used to select 88 extension agents. Data were collected using a questionnaire and analyzed using means, percentages, PPMC, and ordered probit regression. Results show that more than half of the extension agents (52.3%) exhibited unfavorable attitudes towards CSAPs, while 58% moderately participated in their dissemination. Participation was particularly low for water-smart mechanism such as index-based weather insurance ( $\bar{x}=0.00$ ), water harvesting ( $\bar{x}=$ 0.92), drip irrigation ( $\bar{x}=$ 0.73), as well as crop-smart mechanism like integrated pest management ( $\bar{x}=$ 0.62). among rice farmers. Training significantly influenced their attitudes (*p* = 0.011), age (*p* = 0.043), marital status (*p* = 0.028), household size (*p* = 0.026), occupation (*p* = 0.036), years of experience (*p* = 0.004), number of trainings (*p* = 0.035), and attitude (*p* = 0.000) significantly determined their participation levels. The study recommends targeted training and capacity-building initiatives to strengthen extension agents’ attitudes and participation in disseminating CSAPs. Such efforts are essential for strengthening climate resilience, enhancing food security, and promoting dietary diversity through the adoption of sustainable farming systems.

## Introduction

More than half of the world’s population relies on rice for daily sustenance. Over 4 billion people consume it for approximately 21% of their daily calories. In the last five decades, rice production has increased through the interventions of international research centers and governments (1). Sub-Saharan Africa faces challenges like low crop yields and climate change. However, research findings revealed that rice production has the potential to reduce food insecurity in the region (2, 3). This validates the importance of rice research in securing global food systems (1, 4).

Cereal demand in Sub-Saharan Africa is projected to double by 2050, and climate change makes meeting this demand even difficult (5). Rice is very sensitive to shifting weather patterns, and higher temperatures, irregular rainfall, and rising CO_2_ levels negatively impact the yields of rice (6, 7). Furthermore, extreme climate events such as floods and droughts worsen this trend (8).

Climate-smart agricultural practices (CSAPs) provide an innovative strategy to combat the challenges posed by climate change (9). These include the use of improved rice varieties, efficient water management systems, diversified cropping, soil conservation, and climate-based services like weather forecasts and insurance (10, 11). CSAPs improve yields, enhance energy and water efficiency and reduce greenhouse gas emissions. Despite their benefits, adoption in Sub-Saharan Africa remains relatively low (9, 10, 12).

Climate-smart agriculture (CSA) adoption is increasingly recognized as a nutrition-sensitive strategy that can enhance production diversity, improve food access, and lower malnutrition risks, especially in vulnerable regions. This is because CSA practices such as crop rotation, intercropping, agroforestry, and soil fertility management encourage the cultivation of a wider range of crops and livestock. This leads to greater on-farm production diversity, which is strongly associated with higher household dietary diversity scores and improved food consumption patterns in multiple contexts, including Ethiopia, Kenya, South Africa, and West Africa. Furthermore, households adopting combinations of CSA practices often consume more food groups daily, directly supporting more balanced and nutritious diets than their counterparts (13–17).

Agricultural extension agents are key links between research, policy and farmers (18, 19). Their participation in disseminating CSA practices enhances adoption rates, which multiplies nutrition benefits at household and community level. They make up-to-date information available on climate-smart agriculture techniques through visits and advisory services (20, 21). In addition, they help farmers interpret climate information, manage risks and adapt to climate change (22). However, their effectiveness is shaped by their knowledge, attitude, constraints they face (23, 24).

Previous studies have mostly focused on what extension agents know about CSAPs. Less attention has been given to their attitudes and what influences their involvement (19). This study aims to fill that gap. It examines extension agents’ attitudes toward CSAPs, their level of participation, and the factors that affect it. It explores the relationship between attitude and engagement. Evidence from this study will inform extension organizations in motivating extension agents to take an active role. Extension agents’ participation amplifies food security and agricultural yield, while also aiding in better dietary variety and family nutrition, especially in areas susceptible to climate change. CSAPs encourage mixed and climate-resilient cropping systems that boost staple crops as well as other foods. When extension agents assist with the adoption of these practices, they contribute positively to nutrition by diversifying on-farm produce, stabilizing production and enhancing household income for off-farm food purchases. This makes CSAPs dissemination an essential connector linking agricultural extension with food and nutrition security in rural communities. In the end, it aids rice farmers in adapting to climate change and bolsters the resilience of the food system for a rising population.

## Research methodology

### Study area

The research took place in the North Central zone of Nigeria, one of the six geopolitical zones. This North Central area is located within the Guinea savanna region (49). Nevertheless, its vegetation spans across three savannah belts - Guinea, Sudan and Sahel. Consequently, this leads to a prominence of both cereal and root crops in this ecological area. Nigeria’s North Central region encompasses areas such as Plateau, Kwara, Benue, Niger, Kogi, and Nasarawa, along with the Federal Capital Territory (Abuja). This region is positioned between latitudes 7°00′-11°30’ North of the equator and longitudes 4°00′-11°00′East of the Greenwich meridian. The typical yearly rainfall varies from 1,200 mm to 1,500 mm. Temperature remains high for most parts of the year except during harmattan season which starts in November and concludes by February.

### Sampling procedure

In choosing the extension agents, a two-step sampling method was utilized. The initial step was a purposive selection of Kwara, Kogi, and Niger states from among the seven States in North Central Nigeria. This selection stems from these particular States being prominent for their rice cultivation. Stage two involved the selection of all village extension agents, Zonal Extension Officers (ZEOs) and Block Extension Officers (BEOs). In total, 88 were interviewed. This approach was taken because the extension agents mentioned, regardless of their rank, played a role in communicating CSA practices to the farmers. The entire population chosen also reflected their relatively small number. Data were collected using a structured questionnaire.

### Measurement of variables

The extension agents rated their participation in disseminating CSAPs as ‘active’, ‘passive’, or ‘never’, using a scale from 2 to 0. A cumulated mean score was used to categorize into three scale; low, medium and high level of participation.

Participants’ attitudes were measured using 38 items (positive and negative) on a 5-point Likert scale (1 = strongly disagree to 5 = strongly agree). Negative items were reverse-coded, and a summated score was computed for each respondent, producing a possible range of 38–190. This approach is consistent with earlier work demonstrating the validity of composite Likert-type attitude measures (25). This summated score was used as a continuous variable in statistical analysis, including PPMC. To classify respondents, we applied the sample mean score as the cut-off point: For descriptive reporting, respondents scoring above the mean were categorized as having favorable attitudes, while those at or below the mean were categorized as having unfavorable attitudes. This procedure has been used in recent agricultural extension research (26).

Expert validation was conducted, and a pilot test was carried out with 19 extension agents in the study area. Internal consistency of the instrument yielded a Cronbach’s alpha of 0.93, indicating a high reliability. The full list of items is provided in Appendix A.

### Data analysis

The Pearson Product–Moment Correlation was used to assess the relationship between selected socio-economic characteristics and the attitude of the extension agents toward CSAPs.

The ordered probit regression model was employed because the dependent variable is ordinal in nature. Dependent variable: participation in climate-smart agricultural practices (CSAP) was measured on a 3-point Likert scale per item (0 = no participation, 1 = passive participation, 2 = active participation). For the ordered probit analysis, we used a three-category dependent variable: 0 = No/low participation, 1 = Passive/medium participation, 2 = Active /high participation. When multiple items measured participation, we computed the respondent’s mean score across items and classified respondents into low/medium/high using cutoffs (≤1.0 = Low; 1.01–1.5 = Medium; >1.5 = High), which reflect the respondent’s majority behavior across items.

The ordered probit model is typically written as:


$$Y^{*}=\mathrm{\beta X}+\in$$

Where:

$Y^{*}$ is the latent continuous variable (unobserved),

X is a vector of independent variables,

β is the vector of coefficients,

∈ is an error term, assumed to be normally distributed.

The ordinal dependent variable Y is derived from the latent variable $Y^{*}\;$ through thresholds:

If $Y^{*}\;$ ≤ $\theta_{1}$ , then Y = 0,

If $\theta_{1}$ < $Y^{*}$ ≤ $\theta_{2}$ , then Y = 1,

If $\theta_{2}$ < $Y^{*}$ ≤ $\theta_{3}$ ​, then Y = 2,

$Y_{1}$ *= $\beta_{0}$ + $\beta_{1}X_{1i}$ + $\beta_{2}X_{2i}$ + $\beta_{3}X_{3i}$ + $\beta_{4}X_{4i}$ + $\beta_{5}X_{5i}$ + $\beta_{6}X_{6i}$ + $\beta_{7}X_{7i}$ + $\;\beta_{8}X_{8i}+\beta_{9}X_{9i}+\beta_{10}X_{10i}+\beta_{11}X_{11i}+\beta_{12}X_{12i}+\beta_{13}X_{13i}\;e_{i.}$

The explanatory variables (Xs) are as defined:

$X_{1}$ = Kwara State Dummy (1 if the EA works in Kwara State, 0 otherwise)

$X_{2}$ = Kogi State Dummy (1 if the EA works in Kogi State, 0 otherwise)

$X_{3}$ = Age of the agricultural extension agents

$X_{4}$ = Sex of the agricultural extension agents (1 if male, 0 if female)

$X_{5}$ = Marital status of the extension agents (0 = married, 1 = Single)

$X_{6}$ = Household size

$X_{7}$ = Occupation of the extension agents (Primary = 0, Secondary = 1)

$X_{8}$ = Years of experience

$X_{9}$ = Numbers of training

$X_{10}$ = Contacts of agricultural extension agents with research agencies (number of times in the past 1 year)

$X_{11}$ = Ratio of farmers per extension agents

$X_{12}$ = Monthly income of the agricultural extension agents (in Naira)

$X_{13}$ = Attitude of extension agents 1

$X_{14}$ = Educational qualification

$\beta_{s}$ = Parameters to the estimated

$e_{i}$ = Error term

Niger State is the base category. Therefore, the coefficients for Kwara and Kogi States reflect participation likelihood relative to agents from Niger State. *Niger was used as the reference category* because it is the largest state in the sample and provides the most stable baseline.

## Results and discussion

### Attitude of extension agents toward climate smart agricultural practices

The attitude of extension agents toward climate-smart agricultural practices is presented in Table 1. The attitude scores ranged from 105 to 189, with a mean of 157.68 (SD = 17.23). Based on the mean cut-off, respondents scoring above 157.68 were classified as having favorable attitudes, above half (52.3%) of them had an unfavorable attitude toward CSAPs, while less than half (47.7%) of them had a favorable attitude. This suggests that majority of the extension agents have a negative disposition toward CSAPs, and this affects their active performance in disseminating this information to farmers. This is consistent with the findings of Gazi et al. (27) and Hamisu et al. (28) that extension agents who have positive manners and enthusiasm contribute to more effective extension services.

**Table 1:** Attitude of extension agents toward CSA practices.

Categories	Frequencies	Percentage	Mean	SD
Favorable attitude	42	47.7	157.68	17.23
Unfavorable attitude	47	52.3		
Total	88	100.0		

### Participation of extension agents in disseminating CSAPs

Table 2 revealed the distribution of participation of extension agents in CSA dissemination. The result show that the majority of respondents (58.0%) reported medium participation, indicating that most individuals are engaged but not at the highest level. A considerable proportion (30.7%) reported low participation, suggesting that nearly one-third of the sample remains only marginally involved in dissemination processes. In contrast, only 11.4% of respondents reported high participation, reflecting a relatively small group of actively engaged individuals. This limitation not only constrains the scaling of climate-resilient technologies but also increases farmers’ vulnerability to climate-induced risks, and being malnourished. According to Ma & Rahut (29) and Tanti et al. (30), when farmers from marginalized areas adopt CSAPs, it significantly improves their income, productivity and contributes to economic diversification.

**Table 2:** Level of participation in disseminating CSA practices.

CSA dissemination participation levels	Frequency	Percentage	Mean (SD)
Low participation	27	30.7%	1.16 (0.29)
Medium participation	51	58.0%	
High participation	10	11.4%	
Total	88	100%	

**Figure 1:**
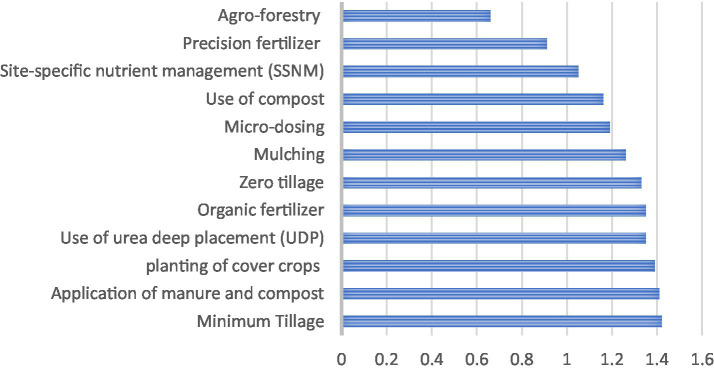
Participation of extension agents in disseminating soil smart mechanism.

The result in Figure 1 showed that a high level of participation was identified in the dissemination of soil smart mechanisms such as site-specific nutrient management (SSNM) ( $\bar{x}=$ 1.05), use of compost ( $\bar{x}=$ 1.16), use of urea deep placement (UDP) ( $\bar{x}=$ 1.35), planting of cover crops ( $\bar{x}=$ 1.39), and minimum tillage ( $\bar{x}=$ 1.42). Farmers in this region are moderately aware of the use of cover crops as a climate change adaptation strategy (31). In the same vein, extension agents in the study were found to have a low level of participation in disseminating information on agroforestry ( $\bar{x}=$ 0.66). This corroborates the findings of Luo et al. (32), Octavia et al. (33), Prajapati et al. (34) that extension agents are more engaged in disseminating basic soil-smart mechanisms than agroforestry in India and Europe, despite the latter’s proven benefits.

**Figure 2:**
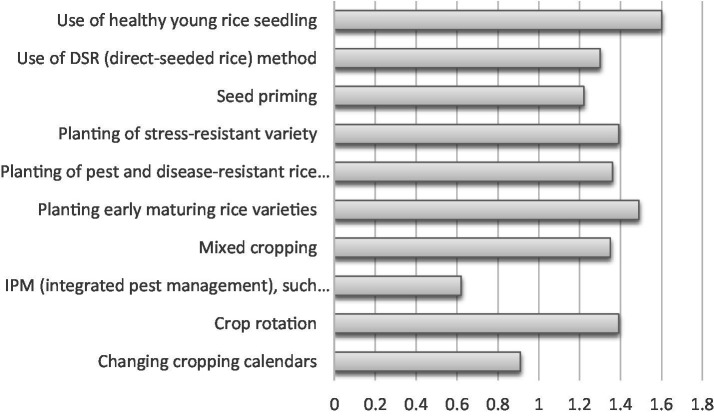
Participation of extension agents in disseminating crop smart mechanism.

Furthermore, the results in Figure 2 illustrated that extension agents participated greatly in disseminating notable crop-smart mechanisms, such as, use of healthy young rice seedlings ( $\bar{x}=$ 1.60), planting early maturing rice varieties ( $\bar{x}=$ 1.49), seed priming ( $\bar{x}=$ 1.22), planting of stress-resistant variety ( $\bar{x}=$ 1.36), crop rotation ( $\bar{x}=$ 1.39) and mixed cropping ( $\bar{x}=$ 1.35). This is consistent with the findings of Dzahan et al. (35) who reported that rural farmers in Benue State indicated the use of mixed cropping, crop rotation, and early planting as adaptation strategies to climate change. The extension agents had a low level of participation in the dissemination of precision agriculture ( $\bar{x}=$ 0.91) and Integrated Pest Management (IPM) ( $\bar{x}=$ 0.62) as CSA practices. This limited participation stems from inadequate training. This align with the findings show that while precision agriculture technologies (e.g., sensors, drones, automated irrigation) are effective, their adoption is limited by cost, technical complexity, and lack of extension agent training. Drawing from Karunathilake et al. (36) and Nwadike et al. (37), there is a very low adoption rate of IPM and precision agriculture among farmers in Nigeria and, and which could be attributed to poor safety knowledge and awareness.

**Figure 3:**
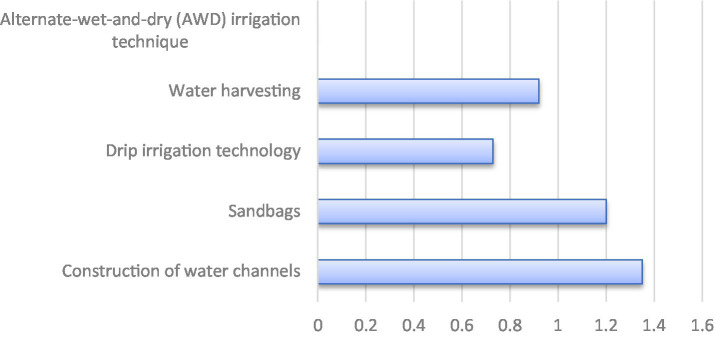
Participation of extension agents in disseminating water smart mechanism.

The fact that the extension agents affirmed a high level of participation in the dissemination of water-smart mechanisms, such as the construction of water channels ( $\bar{x}=$ 1.35) and use of sand bags ( $\bar{x}=$ 1.20) suggests that water smart mechanisms can improve rice production. A clear trend can be seen in Figure 3 presented below. This corroborates the Obaideen et al. (38) that traditional methods like water channels and sandbags are widely promoted in Asia, while Europe promotes the adoption of advanced, automated systems, highlighting the importance of extension agent training and infrastructure for broader technology uptake.

**Figure 4:**
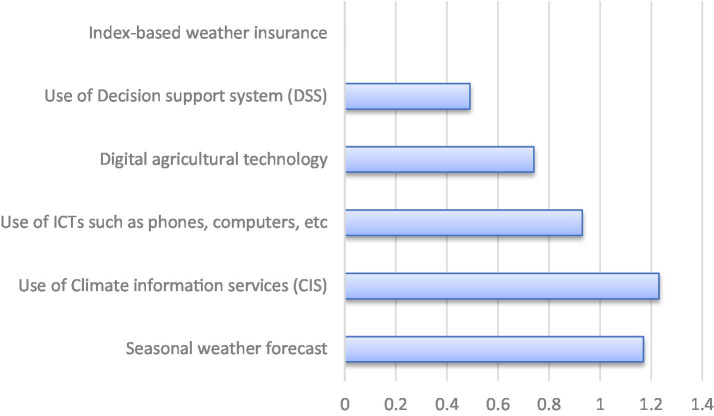
Participation of extension agents in disseminating weather smart mechanism.

Climate education services help farmers to better cope with climate change, which positively improves farm productivity (39). Climate information services ( $\bar{x}=$ 1.23) and seasonal weather forecast ( $\bar{x}=$ 1.17) were highly disseminated by extension agents as weather-smart mechanisms. The result in Figure 4 revealed that they were less involved in disseminating index-based weather insurance ( $\bar{x}=$ 0.00), digital agricultural technology ( $\bar{x}=$ 0.74) and use of ICTs ( $\bar{x}=$ 0.93). This suggests that extension agents effectively share basic weather-smart tools, but advanced mechanisms like agricultural insurance, digital technologies, and ICTs are rarely promoted. This gap could undermine farmers’ long-term climate resilience, highlighting the need for agent training and stronger policy support. This aligns with the report of Madaki and Kaechele (40) that crop farmers in Kogi state, Nigeria, are unaware of agricultural insurance. As well as (41, 42), that advanced digital tools and insurance are rarely mentioned as widely disseminated by extension agents or adopted in North Central, Ethiopia.

**Figure 5:**
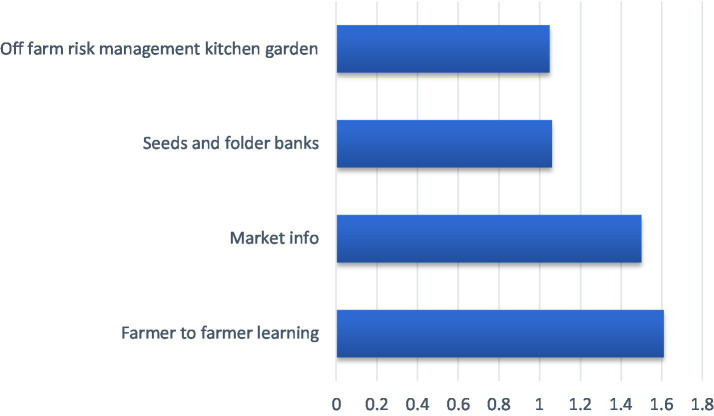
Participation of extension agents in disseminating knowledge smart mechanisms.

Figure 5 showed that the dissemination of knowledge-smart mechanisms was high. The extension agents identified the following practices: farmers-to-farmers learning ( $\bar{x}=$ 1.61), off-farm risk management ( $\bar{x}=$ 1.05), seed banks ( $\bar{x}=$ 1.06) and market information ( $\bar{x}=$ 1.50). This suggests that farmers in the study area engage one another by sharing relevant CSAPs as communicated to them by the extension agents, which in turn boosts their financial life. As highlighted by Madaki et al. (40), farmers’ income and market access are key determinants of climate change strategies.

### Factors influencing participation of extension agents in disseminating CSA practices

Table 3 displays the results of ordered probit regression analysis examining factors influencing the participation of agricultural extension agents. The result shows that the age of the extension agent is marginally significant (*p* = 0.043*) in influencing their participation in CSA dissemination. Specifically, older extension agents are associated with a slightly higher likelihood of low participation but the effect is weak and only marginally significant. This could suggest that younger agents might be more open or better equipped to actively engage in CSA practices, while older agents may have less motivation or fewer resources to engage in the dissemination. This corroborates with the findings of Jones et al. (43) in Ghana.

**Table 3:** Ordered probit marginal effects on determinants of extension agents’ participation.

Variable	Marginal effect (low participation)	Marginal effect (medium participation)	Marginal effect (high participation)	*p*-value
Kwara state	0.0231 (0.2329)	0.0003 (0.0246)	0.0029 (0.2183)	0.989
Kogi state	−0.0148 (0.2410)	0.0003 (0.0260)	0.0133 (0.2153)	0.951
Age	0.0094 (0.0047)	−0.0010 (0.0026)	−0.0085 (0.0045)	0.043**
Sex	−0.0196 (0.0837)	0.0021 (0.0099)	0.0175 (0.0754)	0.815
Marital status	0.5891 (0.2681)	−0.0622 (0.1692)	−0.5269 (0.2254)	0.028**
Household size	−0.0141 (0.0064)	0.0015 (0.0037)	0.0126 (0.0060)	0.026**
Occupation	−0.2001 (0.0956)	0.0211 (0.0562)	0.1790 (0.0883)	0.036**
Years of experience	0.0131 (0.0045)	−0.0009 (0.0038)	−0.0117 (0.0034)	0.004***
Numbers of training	−0.0195 (0.0093)	0.0021 (0.0057)	0.0175 (0.0086)	0.035**
Contact with agency	−0.0008 (0.0021)	0.0001 (0.0003)	0.0007 (0.0019)	0.683
Ratio of farmers/extension agents	−2.17e-06 (2.75e-06)	1.42e-07 (6.62e-07)	1.94e-06 (2.48e-06)	0.434
Monthly income	2.02e-07 (4.04e-07)	−1.73e-08 (7.81e-08)	−1.80e-07 (3.63e-07)	0.617
Attitude of extension agents	−0.0087 (0.0021)	0.0009 (0.0024)	0.0078 (0.0021)	0.000***
Educational qualification	0.0977 (0.0706)	−0.0103 (0.0259)	−0.0874 (0.0703)	0.166

LR *χ*^2^(25):99.90, Prob > *χ*^2^:0.0000, log likelihood: −31.5203. *p* < 0.001***, *p* < 0.05**, *p* < 0.10*.

The marital status of an extension agent significantly affects their level of participation (*p* = 0.028**). Single agents are more likely to engage in medium or high participation in CSA practices compared to their married counterparts. This could be due to the potential flexibility that single extension agents might have in their schedules, which could allow for more active engagement in the dissemination of CSA practices, as opposed to married agents who may have more family responsibilities. Furthermore, the household size of extension agents is significantly related to their participation in CSA dissemination (*p* = 0.026**). In similar vein, larger household sizes are positively correlated with higher participation in CSA dissemination. This might suggest that extension agents with larger families may feel a stronger obligation to engage in more sustainable practices like CSA, potentially due to a greater awareness of environmental impacts or the importance of resource conservation for future generations. This aligns with (44, 45) that larger household size enhances participation in CSA dissemination, primarily due to increased labor and a stronger drive for sustainable resource management.

The result further reveals that extension agents with secondary occupations (i.e., those who combine extension work with other income-generating activities) were significantly more likely to participate actively in CSA dissemination compared to those whose primary occupation was extension work (*p* = 0.036). At first glance, this result may seem counterintuitive, since one might expect full-time extension agents to be more engaged in dissemination, however, it is possible that agents with additional livelihoods may draw on broader social networks, and alternative livelihood experiences that they can leverage to enhance their engagement in CSA dissemination.

The table also reveals that with more years of experience are significantly more likely to be in higher participation categories (*p* = 0.004***), suggesting that experienced agents are more involved in CSA practice dissemination. This is because more experienced extension agents are likely to have better knowledge, skills, and networks to promote CSA practices. They are also more likely to have the confidence and reputation needed to influence farmers’ participation in such practices. This aligns with (18, 21). Similarly, the number of trainings an extension agent has participated in is significantly associated with higher participation in CSA practices (*p* = 0.035**), indicating that training plays an important role in preparing agents to actively disseminate CSA practices.

Finally, the attitude of extension agents (*p* = 0.000***), plays a vital role in influencing their participation. A positive attitude toward a profession is associated with increased professional motivation or institutional motivation (46).

**Table 4:** PPMC analysis of the socio-economic characteristics and attitude of extension agents toward CSAPs.

Variable	*r*-value	*p*-value
Age	−0.042	0.697
Educational qualification	−0.047	0.664
Years of experience	−0.124	0.248
Number of trainings	0.271	0.011^***^

^***^Significant at 1%.

### Socio-economic characteristics and attitude of extension agents

The Pearson product–moment analysis revealed that there is a positive and statistically significant linear relationship between the attitude of agricultural extension agents and the number of trainings they received on CSA practices (*r* = 0.271; *p* < 0.05). This explains that agricultural extension agents who receive more training tend to have a more positive or enhanced attitude toward their work or role in disseminating CSAPs. This aligns with (47, 48) that there is a linkage between training, development and motivation to work. While training correlates with better attitudes, structural constraints may still limit participation. Hence, Periodic training and incentives (e.g., awards, promotions) are recommended to sustain motivation and performance (21) (Table 4).

## Conclusion and recommendation

This study revealed that extension agents in the study area generally held unfavorable attitudes toward climate-smart agricultural practices (CSAPs) and showed only moderate participation in their dissemination. Attitudes were strongly influenced by training, while participation was shaped by age, marital status, household size, occupation, numbers of training, years of experience, and attitudes. These gaps in capacity and motivation linked to inadequate training and weak institutional support, limit CSAP adoption and, by extension, reduce farmers’ resilience, productivity, and nutrition security.

Policy responses should therefore address both human capacity and institutional barriers. Government agencies should expand structured, recurrent training and invest in digital platforms, climate services, and insurance-linked advisory tools. Extension organizations should adopt age-inclusive strategies, by leveraging the innovative potential of younger agents, provide retraining for older agents, and introduce workload flexibility to enable active participation across marital and household categories. Research institutions should co-develop training curricula with extension services and ensure timely transfer of CSAP innovations. Finally, development partners can strengthen outreach through digital infrastructure and support programs that integrate socio-demographic realities into extension delivery.

By tailoring policies to the diverse characteristics of agents, the extension system can more effectively disseminate CSAPs, thereby accelerating adoption, improving food security, and advancing climate-resilient, nutrition-sensitive food systems.

### Limitation of the study

This study is limited by its cross-sectional design, the relatively narrow geographic coverage, and its reliance on self-reported data, which may be subject to response bias. Future research should consider longitudinal and mixed-methods approaches to better establish causal relationships and provide deeper insights into extension agents’ engagement with climate-smart agriculture.

## Data Availability

The raw data supporting the conclusions of this article will be made available by the authors, without undue reservation.

## References

[1] Mishra AK Pede VO Arouna A Labarta R Andrade R Veettil PC. Helping feed the world with rice innovations: CGIAR research adoption and socioeconomic impact on farmers. Glob Food Sec. (2022) 33:100628. doi: 10.1016/J.GFS.2022.100628 35784265 PMC9231551

[2] Wudil AH Ali A Aderinoye-Abdulwahab S Raza HA Mehmood HZ Sannoh AB. Determinants of food security in Nigeria: empirical evidence from beneficiaries and non-beneficiaries rice farmers of the Kano River irrigation project. Front Sustain Food Syst. (2023) 7:999932. doi: 10.3389/fsufs.2023.999932

[3] Wudil AH Usman M Rosak-Szyrocka J Pilař L Boye M. Reversing years for global food security: a review of the food security situation in sub-Saharan Africa (SSA). Int J Environ Res Public Health. (2022) 19:14836. doi: 10.3390/ijerph192214836 36429555 PMC9690952

[4] Rodenburg J Saito K. Towards sustainable productivity enhancement of rice-based farming systems in sub-Saharan Africa. Field Crop Res. (2022) 287:108670. doi: 10.1016/J.FCR.2022.108670

[5] Alimagham S Van Loon MP Ramirez-Villegas J Adjei-Nsiah S Baijukya F Bala A. Climate change impact and adaptation of rainfed cereal crops in sub-Saharan Africa. Eur. J. Agron. (2024) 155:127137. doi: 10.1016/J.EJA.2024.127137

[6] Mukhopadhyay D Das D. Impact of climate change on rice production in African countries: a panel data analysis. Afr J Food Agric Nutr Dev. (2023) 23:23525–46. doi: 10.18697/ajfand.120.22275

[7] Stuch B Alcamo J Schaldach R. Projected climate change impacts on mean and year-to-year variability of yield of key smallholder crops in sub-Saharan Africa. Clim Dev. (2021) 13:268–82. doi: 10.1080/17565529.2020.1760771

[8] Akpa AF. The effects of climate extreme events on selected food crop yields in sub-Saharan Africa. Heliyon. (2024) 10:e30796. doi: 10.1016/J.HELIYON.2024.E30796 38756606 PMC11096961

[9] Ogisi OD Begho T. Adoption of climate-smart agricultural practices in sub-Saharan Africa: a review of the progress, barriers, gender differences and recommendations. Farming Syst. (2023) 1:100019. doi: 10.1016/J.FARSYS.2023.100019

[10] Bijarniya D Parihar CM Jat RK Kalvania K Kakraliya SK Jat ML. Portfolios of climate smart agriculture practices in smallholder Rice-wheat system of eastern indo-Gangetic Plains—crop productivity, resource use efficiency and environmental foot prints. Agronomy. (2020) 10:1561. doi: 10.3390/agronomy10101561

[11] Pandeya S Gajurel A Mishra BP Devkota K Gyawali BR Upadhaya S. Determinants of climate-smart agriculture adoption among Rice farmers: enhancing sustainability. Sustainability. (2024) 16:10247. doi: 10.3390/su162310247

[12] Habib-ur-Rahman M Ahmad A Raza A Hasnain MU Alharby HF Alzahrani YM. Impact of climate change on agricultural production; issues, challenges, and opportunities in Asia. Front Plant Sci. (2022) 13:925548. doi: 10.3389/fpls.2022.925548 PMC9621323 36325567

[13] Khumalo NZ Sibanda M Mdoda L. The effect of heterogeneous adoption of climate-smart agriculture practices on household food and nutrition security of small-scale urban crop farmers in eThekwini municipality. PLoS Clim. (2025) 4:e0000551. doi: 10.1371/journal.pclm.0000551 PMC13235438 42253735

[14] Mwalupaso GE Eshetie AM Matafwali E Akter A Lu H Geng X. Rethinking household food security under a changing climate in drought prone areas of Ethiopia. Land Use Policy. (2025) 150:107437. doi: 10.1016/j.landusepol.2024.107437

[15] Omotoso AB Omotayo AO. Impact of behavioural intention to adopt climate-smart agricultural practices on the food and nutrition security of farming households: a microeconomic level evidence. Clim Chang. (2024) 177:117. doi: 10.1007/s10584-024-03775-6

[16] Santalucia S Sibhatu KT. Nourishing the farms, nourishing the plates: association of climate-smart agricultural practices with household dietary diversity and food security in smallholders. Agribusiness. (2024) 40:513–33. doi: 10.1002/agr.21892

[17] Wekesa BM Ayuya OI Lagat JK. Effect of climate-smart agricultural practices on household food security in smallholder production systems: micro-level evidence from Kenya. Agric Food Secur. (2018) 7:1–14. doi: 10.1186/s40066-018-0230-0

[18] Maka L Ngotho T Walker S Ngcamphalala S Maboa L. An assessment of climate-smart agriculture (CSA) practices skills amongst extension practitioners in South Africa. South African J Agric Exten (SAJAE). (2021) 49:70–83. doi: 10.17159/2413-3221/2021/v49n2a12802

[19] Olorunfemi OD Olorunfemi TO Oladele OI Malomo JO. Knowledge of extension agents on c limate smart agricultural initiatives in south-West Nigeria. J Agric Exten. (2021) 25:22–30. doi: 10.4314/jae.v25i4.3

[20] Kumar A Malik J Kamboj M. Relevance of extension advisory services in climate smart agriculture: a review. Mausam. (2022) 73:695–704. doi: 10.54302/mausam.v73i3.5937

[21] Ojo IE Akangbe JA Kolawole EA Owolabi AO Obaniyi KS Ayeni MD. Constraints limiting the effectiveness of extension agents in disseminating climate-smart agricultural practices among rice farmers in north-Central Nigeria. Front Clim. (2024) 6:1297225. doi: 10.3389/fclim.2024.1297225

[22] Ojo IE Akangbe JA Owolabi AO. Needs of extension agents on techniques for climate-smart Rice production in north-central. Nigeria J Agric Exten. (2023) 28:86–92. doi: 10.4314/jae.v28i1.12S

[23] Abegunde VO Obi A. The role and perspective of climate smart agriculture in Africa: a scientific review. Sustainability. (2022) 14:2317. doi: 10.3390/su14042317

[24] Ozioko RI Eze KC Emordi AN Okoronkwo DJ Nwobodo CE. Capability of extension agents in disseminating climate change information in Delta state Nigeria. J Agric Exten. (2022) 26:74–85. doi: 10.4314/jae.v26i3.7

[25] Dutton WH Blum MP. The measurement of attitudes toward arithmetic with a Likert-type test. Elem Sch J. (1968) 68:259–64. doi: 10.1086/460443

[26] Saba S Saini H. A tool to measure attitude of agriculture students towards student-ready Programme in employability generation. Gujarat J Exten Educ. (2023) 36:113–8. doi: 10.56572/gjoee.2023.36.1.0022

[27] Gazi MAI Yusof MF Islam MA Amin MB Senathirajah AR b S. Analyzing the impact of employee job satisfaction on their job behavior in the industrial setting: an analysis from the perspective of job performance. J Open Innov: Technol Mark Complex. (2024) 10:100427. doi: 10.1016/J.JOITMC.2024.100427

[28] Hamisu S Umar S Oladosu IO Gona A. Assessment of job behavior of agricultural extension workers: a case study of SAFE Programme beneficiaries in North-Western Nigeria. Asian J Adv Res Rep. (2020) 10:1–7. doi: 10.9734/ajarr/2020/v10i230236

[29] Ma W Rahut DB. Climate-smart agriculture: adoption, impacts, and implications for sustainable development. Mitig Adapt Strateg Glob Chang. (2024) 29:44. doi: 10.1007/s11027-024-10139-z

[30] Tanti PC Jena PR Timilsina RR Rahut DB. Enhancing crop yields and farm income through climate-smart agricultural practices in eastern India. Mitig Adapt Strateg Glob Chang. (2024) 29:35. doi: 10.1007/s11027-024-10122-8

[31] Ayeni MD Owolabi A Ayeni OT Alhassan YJ. Climate smart agriculture strategies among crop farmers in north Central Nigeria: implication on farm productivity Proc. Int. Conf. Sci. Eng. Bus. Sustain. Dev. Goals (SEB-SDG) Piscataway, NJ: IEEE. 1–8. doi: 10.1109/SEB-SDG57117.2023.10124616

[32] Luo Z Zhang S Zhao Z Minasny B Chang J Huang J. Soil-smart cropping for climate-smart production. Geoderma. (2024) 451:117061. doi: 10.1016/j.geoderma.2024.117061

[33] Octavia D Murniati SS Hani A Mindawati N Suratman SD Junaedi A. Smart agroforestry for sustaining soil fertility and community livelihood. For Sci Technol. (2023) 19:315–28. doi: 10.1080/21580103.2023.2269970

[34] Prajapati CS Priya NK Bishnoi S Vishwakarma SK Buvaneswari K Shastri S. The role of participatory approaches in modern agricultural extension: bridging knowledge gaps for sustainable farming practices. J Exp Agric Intern. (2025) 47:204–22. doi: 10.9734/jeai/2025/v47i23281

[35] Dzahan HL Beeior CT Ogbu ES Igbanyam C. Awareness and adaptation strategies to climate change among rural farmers in Ukum local government area of Benue state. Nigerian J Agric Agricul Technol. (2024) 4:234–42. doi: 10.59331/njaat.v4i4A.879

[36] Karunathilake EMBM Le AT Heo S Chung YS Mansoor S. The path to smart farming: innovations and opportunities in precision agriculture. Agriculture. (2023) 13:1593. doi: 10.3390/agriculture13081593

[37] Nwadike C Joshua VI Doka PJS Ajaj R Abubakar Hashidu U Gwary-Moda S. Occupational safety knowledge, attitude, and practice among farmers in northern Nigeria during pesticide application—a case study. Sustainability. (2021) 13:10107. doi: 10.3390/su131810107

[38] Obaideen K Yousef BA AlMallahi MN Tan YC Mahmoud M Jaber H. An overview of smart irrigation systems using IoT. Energy Nexus. (2022) 7:100124. doi: 10.1016/j.nexus.2022.100124

[39] Eneji CVO Onnoghen NU Acha JO Diwa JB. Climate change awareness, environmental education and gender role burdens among rural farmers of northern Cross River state, Nigeria. Int J Clim Change Strateg Manag. (2021) 13:397–415. doi: 10.1108/IJCCSM-06-2020-0070

[40] Madaki MY Kaechele H Bavorova M. Agricultural insurance as a climate risk adaptation strategy in developing countries: a case of Nigeria. Clim Pol. (2023) 23:747–62. doi: 10.1080/14693062.2023.2220672

[41] Asfaw A Simane B Bantider A Hassen A. Determinants in the adoption of climate change adaptation strategies: evidence from rainfed-dependent smallholder farmers in north-Central Ethiopia (Woleka sub-basin). Environ Dev Sustain. (2019) 21:2535–65. doi: 10.1007/s10668-018-0150-y

[42] Gashure S. Adaptation strategies of smallholder farmers to climate variability and change in Konso. Ethiopia Sci Rep. (2024) 14:19203. doi: 10.1038/s41598-024-70047-9 39160267 PMC11333751

[43] Jones EO Tham-Agyekum EK Ankuyi F Ankrah DA Akaba S Shafiwu AB. Mobile agricultural extension delivery and climate-smart agricultural practices in a time of a pandemic: evidence from southern Ghana. Environ Sustain Indic. (2023) 19:100274. doi: 10.1016/j.indic.2023.100274

[44] Naazie GK Agyemang I Tampah-Naah AM. Our cities, our farm lands: the socioeconomic determinants of urban households participation in urban agricultural production under climatic stressors. Heliyon. (2024) 10:e35539. doi: 10.1016/j.heliyon.2024.e35539 39224284 PMC11366882

[45] Zakaria A Azumah SB Appiah-Twumasi M Dagunga G. Adoption of climate-smart agricultural practices among farm households in Ghana: the role of farmer participation in training programmes. Technol Soc. (2020) 63:101338. doi: 10.1016/j.techsoc.2020.101338

[46] Tafida I Yusuf AK Kabir AM Abdullahi A. Assessment of training needs and competence level of extension Workers in Kano State. Nigeria FUDMA J Sci. (2021) 5:424–35. doi: 10.33003/fjs-2021-0501-587

[47] Dabiah A. T. Alotibi Y. S. Herab A. H. (2023). Attitudes of agricultural extension workers toward the use of electronic extension methods in agricultural extension in the Kingdom of Saudi Arabia.

[48] Suhardi AR Oktari SD Budiawan A. The influence of training programs and motivation on employee work productivity. Int J Sci Soc. (2023) 5:887–96. doi: 10.54783/ijsoc.v5i4.876

[49] National Bureau of Statistics (NBS). (2025). Annual Abstract of Statistics. Abuja, Nigeria: Federal Republic of Nigeria.

