# Supplementary material for: Extension agents’ attitudes and participation in disseminating climate-smart agricultural practices in North-Central, Nigeria
Source: Front Nutr. 2025 Sep 18;12:1663720. doi: 10.3389/fnut.2025.1663720 (PMC12490237; doi:10.3389/fnut.2025.1663720)
Supplement: Supplementary file 1 [file Supplementary_file_1.docx]

**APPENDIX A: Attitude of Extension Agents Towards Disseminating CSA**

| S/N | Attitudinal statement | Strongly agree | Agree | Undecided | Disagree | Strongly disagree |
| --- | --- | --- | --- | --- | --- | --- |
|  | Usefulness of CSAPs |  |  |  |  |  |
| 1. | CSAPs are useful for efficiently increasing rice yield. |  |  |  |  |  |
| 2. | CSAPs are a valuable alternative for farmers to adapt to and mitigate the effects of climate change. |  |  |  |  |  |
| 3. | CSAPs are a useful tool for ensuring effective and efficient farming. |  |  |  |  |  |
| 4. | CSAPs are a practical method for helping farmers cope with environmental conditions/climate change. |  |  |  |  |  |
| 5. | CSAPs provide a useful platform for accessing current information on crops (rice) marketing. |  |  |  |  |  |
| 6. | Educating farmers on certain CSAPs is useful in helping reduce yield loss. |  |  |  |  |  |
| 7. | CSAPs are not useful for efficiently increasing rice yield. |  |  |  |  |  |
| 8. | CSAPs are not a useful tool for ensuring effective and efficient farming |  |  |  |  |  |
| 9. | CSAPs are not a practical method for helping farmers cope with environmental conditions/climate change. |  |  |  |  |  |
| 10. | CSAPs are not a useful platform for accessing current information on rice marketing. |  |  |  |  |  |
| 11. | Educating farmers on certain CSAPs is not useful in helping reduce yield loss. |  |  |  |  |  |
| 12. | I agreed with building extension agents’ capability on CSAPs. |  |  |  |  |  |
| 13. | I have a vital role to play in disseminating CSAPs |  |  |  |  |  |
| 14. | My skills and knowledge as an extension agent on CSAPs need to be frequently improved via training. |  |  |  |  |  |
| 15. | I have confidence in myself when teaching the farmers' CSAPs needed for their production. |  |  |  |  |  |
| 16. | I don’t agree with building extension agents’ capability on CSAPs. training. |  |  |  |  |  |
| 17 | I do not have a crucial role to play in disseminating CSAPs. |  |  |  |  |  |
| 18. | My skills and knowledge as an extension agent on CSAPs do not need to be frequently improved via training. |  |  |  |  |  |
| 19. | I am not confident in myself when teaching the farmers CSAPs needed for their production. |  |  |  |  |  |
| 20 | I believe with providing farmers with CSAPs manuals so as to aid easy learning. |  |  |  |  |  |
| 21 | I enjoy participating in enhancing farmers' capacity through CSAPs, as it increases their income. |  |  |  |  |  |
| 22 | . It is very important to have a local leader with adequate knowledge of CSAPs so as to ensure follow-up on how the CSA practices are being carried out. |  |  |  |  |  |
| 23 | CSAPs should be included in and taught with different audio-visual aids to achieve better understanding |  |  |  |  |  |
| 24 | It is very important to ensure adequate involvement of farmers while teaching them, as CSAPs is more effective compared to the traditional method of rice farming. |  |  |  |  |  |
| 25 | It is very important to practically demonstrate CSAPs to farmers on their fields based on their needs. |  |  |  |  |  |
| 26 | It is appropriate to teach and implement CSAPs programmes in rural areas.. |  |  |  |  |  |
| 27 | I am not interested with providing farmers with CSAPs manuals so as to aid easy learning. |  |  |  |  |  |
| 28 | I am not interested participating in activities that enhance farmers’ capacity through CSAPs |  |  |  |  |  |
| 29 | It is not crucial to have a local leader with adequate knowledge of CSAPs so as to ensure follow-up on how the CSA practices are being carried out.. farming. |  |  |  |  |  |
| 30 | CSAPs should not be included and taught with different audio-visual aids . |  |  |  |  |  |
| 31 | I don’t need to provide farmers with CSAPs manuals so as to aid easy learning. |  |  |  |  |  |
| 32 | It is not important to practically demonstrate CSAPs to farmers |  |  |  |  |  |
| 33 | It is not important to ensure adequate involvement of farmers while teaching them as CSAP is more effective compared to the traditional method of rice farming. |  |  |  |  |  |
| 34 | Lack of follow-up support after training makes CSAPs less effective for farmers. |  |  |  |  |  |
| 35 | The output of farmers is expected to increase when I disseminate CSAPs to them. |  |  |  |  |  |
| 36 | Limited availability of CSAPs-related equipment and materials reduces farmers’ ability to use them. |  |  |  |  |  |
| 37. | I do not support CSAPs as an appropriate method for farmers to cope with environmental conditions or climate change. |  |  |  |  |  |
| 38. | Lack of access to credit prevents farmers from implementing CSAPs effectively. |  |  |  |  |  |

**APPENDIX B:**  **Marginal Effects from Ordered Logit Regression on Participation Categories**

| **Variable** | **Marginal Effect (Category 0)** | **Marginal Effect (Category 1)** | **Marginal Effect (Category 2)** | **P-Value** |
| --- | --- | --- | --- | --- |
| Kwara state | 0.0231 (0.2348) | -0.0046 (0.2728) | -0.0215 (0.2185) | 0.922 |
| Kogi state | -0.0046 (0.2728) | 0.0003 (0.0189) | 0.0133 (0.2539) | 0.987 |
| Age | 0.0085 (0.0051) | -0.0006 (0.0026) | -0.0080 (0.0048) | 0.097* |
| Sex | -0.0295 (0.0822) | 0.0020 (0.0098) | 0.0275 (0.0780) | 0.720 |
| Marital status | 0.5869 (0.2991) | -0.0404 (0.1809) | -0.5465 (0.2677) | 0.050** |
| Household size | -0.0122 (0.0069) | 0.0008 (0.0037) | 0.0114 (0.0065) | 0.026** |
| Occupation | -0.1921 (0.0981) | 0.0211 (0.0586) | 0.1790 (0.0931) | 0.050** |
| Years of experience | 0.0135 (0.0049) | -0.0009 (0.0042) | -0.0126 (0.0034) | 0.006*** |
| Numbers of training | -0.0188 (0.0097) | 0.0021 (0.0057) | 0.0175 (0.0086) | 0.035** |
| Contact with agency | -0.0008 (0.0020) | 0.0001 (0.0003) | 0.0007 (0.0019) | 0.683 |
| Ratio of farmers/ extension agents | -2.17e-06 (2.75e-06) | 1.42e-07 (6.62e-07) | 1.94e-06 (2.48e-06) | 0.434 |
| Monthly income | 2.02e-07 (4.04e-07) | -1.73e-08 (7.81e-08) | -1.80e-07 (3.63e-07) | 0.617 |
| Attitude of extension agents | -0.0088 (0.0022) | 0.0006 (0.0027) | 0.0078 (0.0021) | 0.000*** |
| Educational qualification | 0.0977 (0.0706) | -0.0103 (0.0259) | -0.0874 (0.0703) | 0.166 |

**LR chi2(25):99.39 Prob > chi2: 0.0000 Log Likelihood:-31.7740**

p <0.001***, p <0.05**, p <0.10*
